# End-of-Fiber Signals Strongly Influence the First and Second Phases of the M Wave in the *Vastus Lateralis*: Implications for the Study of Muscle Excitability

**DOI:** 10.3389/fphys.2018.00162

**Published:** 2018-03-08

**Authors:** Javier Rodriguez-Falces, Nicolas Place

**Affiliations:** ^1^Department of Electrical and Electronical Engineering, Public University of Navarra, Pamplona, Spain; ^2^Institute of Sport Sciences, University of Lausanne, Lausanne, Switzerland

**Keywords:** compound muscle action potential, end-of-fiber signals, pennate muscles, muscle excitability, fascicle length, non-propagating components, extracellular potential generation

## Abstract

It has been recurrently observed that, for compound muscle action potentials (M wave) recorded over the innervation zone of the *vastus lateralis*, the descending portion of the first phase generally shows an “inflection” or “shoulder.” We sought to clarify the electrical origin of this shoulder-like feature and examine its implications. M waves evoked by maximal single shocks to the femoral nerve were recorded in monopolar and bipolar configurations from 126 individuals using classical (10-mm recording diameter, 20-mm inter-electrode distance) electrodes and from eight individuals using small electrodes arranged in a linear array. The changes of the M-wave waveform at different positions along the muscle fibers' direction were examined. The shoulder was identified more frequently in monopolar (97%) than in bipolar (46%) M waves. The shoulder of M waves recorded at different distances from the innervation zone had the same latency. Furthermore, the shoulder of the M wave recorded over the innervation zone coincided in latency with the positive peak of that recorded beyond the muscle. The positive phase of the M wave detected 20 mm away from the innervation zone was essentially composed of non-propagating components. The shoulder-like feature in monopolar and bipolar M waves results from the termination of action potentials at the superficial aponeurosis of the *vastus lateralis*. We conclude that, only the amplitude of the first phase, and not the second, of M waves recorded monopolarly and/or bipolarly in close proximity to the innervation zone can be used reliably to monitor possible changes in muscle membrane excitability.

## Introduction

**Figure 1:**
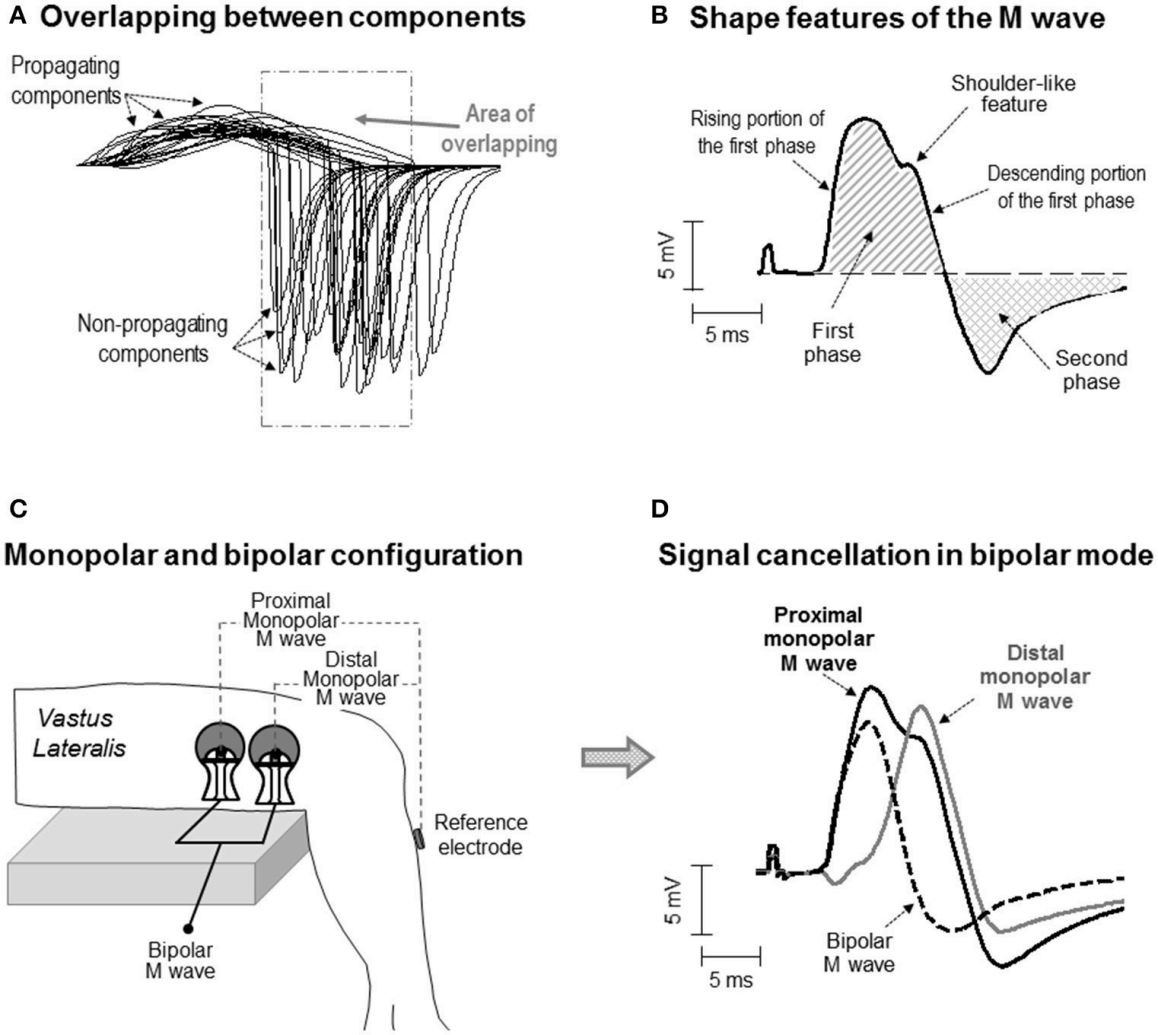
**(A)** Simulation of some of the single fiber action potentials constituting an M wave for an electrode located at 25 mm from the innervation zone. Note the overlapping between the propagating and non-propagating components of the different single fiber potentials. **(B)** Definition of some waveform features of the M wave. Note that the M wave exhibits a shoulder in the descending portion of the first phase. **(C)** Schematic representation of the electrode settings used to record surface EMG activity in monopolar and bipolar configuration in the *vastus lateralis*. **(D)** Representative example of a bipolar M wave obtained by subtracting the distal from the proximal monopolar M wave. Note that part of the electrical content captured by the electrode is lost when the monopolar signals are subtracted and that, as a result, some of the features of the monopolar M wave (i.e., the shoulder) are not present in the bipolar M wave.

In the last decades, several investigations have advanced the knowledge on the electrical generation (electrogenesis) of the compound muscle action potential (M wave) (Gydikov and Kosarov, 1972; Lateva et al., 1996; Nandedkar and Barkhaus, 2007). However, to date, there is not a complete understanding of the way in which the shape of the M wave is determined by the electrical activity in the muscle (Lateva and McGill, 1998). The basic biphasic morphology of the M wave is normally explained by saying that the first phase mainly results from the propagation of action potentials along the muscle fibers, whereas the second phase reflects the extinction of these action potentials at the tendon (Lateva et al., 1996). However, the practice of identifying the first and second phases of the M wave with the *propagating* and *non-propagating* components, respectively, is a simplification of reality, as there are several factors adding complexity to the formation of the M-wave waveform. First, the issue of overlapping: because the individual constituents of an M wave are dispersed in time, the *propagating* component of some fibers inevitably overlaps with the *non-propagating* component of other fibers (Figure 1A). The overlapping phases diminish each other due to phase cancelation (Keenan et al., 2006). Moreover, the degree of overlapping between the *propagating* and *non-propagating* components increases as the electrode is moved away from the innervation zone (Rodriguez-Falces and Place, 2014). Therefore, a clear visual distinction of the *propagating* and *non-propagating* signals in the M-wave waveform may not be possible in all cases.

An additional factor influencing the electrical formation of the M wave is the arrangement of muscle fibers: parallel or oblique to the force-generating axis. In pennate muscles, due to the inclination of the fibers relative to the skin surface, the electrical activity of the most superficial (close to the skin) portion of the fiber makes the greatest contribution to the signal recorded by the electrode. The result is that, in pennate muscles, surface potentials have a more localized spatial distribution (Mesin et al., 2011), implying that the peak of the *propagating* component may be in close temporal proximity to the *non-propagating* component. In these circumstances, the non-propagating components might contribute significantly to both the first and second phases of the M wave. Therefore, it might be that, in pennate muscles, the first phase of an M wave is frequently contaminated by the signals arising from the extinction of action potentials. This contamination might be revealed by the presence of a “distinct” deformation (in the form of a shoulder) in the first phase (Figure 1B). The current work explores this possibility.

The impetus for the present study came from the observation that most of the M waves recorded in the *vastus lateralis* and *tibialis anterior* under monopolar configuration showed an “inflection” or shoulder-like feature in the descending portion of the first phase (for examples, see Figure 1B and also Rodriguez-Falces et al., 2015; Rodriguez-Falces and Place, 2016). While this morphologic feature has not been reported in the *vastus lateralis* by previous authors (presumably because they all used a bipolar configuration), it was already noticed in the *tibialis anterior* by Thomas et al. (1989), who adopted a monopolar configuration. Unfortunately, Thomas and colleagues did not comment on the nature and possible significance of the shoulder. Clearly, more research is needed to establish whether the shoulder-like feature is related to the action potential extinction at the tendon or is a natural consequence of the propagating action potentials.

To investigate the electrical origin of the shoulder-like feature, a monopolar electrode configuration must be adopted (Figure 1C). The reason is that monopolar signals contain the entire informative content of the action potential propagation and extinction (Tucker and Türker, 2005). However, in some muscles, such as the *quadriceps*, the bipolar electrode arrangement is commonly adopted due to its better cross-talk rejection (Merletti and Hermens, 2004). The problem inherent to the bipolar montage is that part of the electrical signal is lost when the contributions from the proximal and distal electrodes are subtracted (Figure 1D). The implication is that the shape of the bipolarly-recorded M wave cannot be expected to reflect reliably the *genuine* electrical activity generated by the propagation and extinction of the action potentials (Figure 1D). Therefore, the possible interference of the *non-propagating* components on the M-wave first phase cannot be studied properly using the bipolar configuration.

The importance of identifying the parts of the M wave that correspond to the propagation and extinction events is that only the propagating portion of the compound potential reliably reflects the changes in membrane excitability (Rodriguez-Falces and Place, 2016). Indeed, non-propagating components can be misleading as indicators of membrane excitability since these components depend on many uncontrollable factors other than membrane properties. For example, *end-of-fiber* components are highly sensitive to positional changes of the tendon relative to the recording electrodes and, hence, any change in the muscle architectural properties could drastically influence the magnitude of these components (Rodriguez-Falces and Place, 2014). Therefore, it seems necessary to determine whether, and the extent to which, the first phase of an M wave is contaminated by *end-of-fiber* components, as this may compromise the usefulness of this phase as an index of membrane excitability.

The objective of the present study was to determine the electrical origin of the shoulder-like feature observable in the first phase of M waves recorded in pennate muscles. It was hypothesized that the shoulder is not due to electrical activity propagating under the electrode, but the result of the extinction of action potentials. This work included data from 126 individuals in order to establish the frequency of appearance of the shoulder in monopolar and bipolar M waves. This study was designed to gain insight into the electrophysiolgical determinants of the M wave in pennate muscles, with a view to determining which parts of the monopolar and bipolar M waves can be used to reliably study membrane excitability.

## Materials and methods

### Organization of the study and participants

To investigate the origin and characteristics of the shoulder-like feature in the M waves of pennate muscles, four main steps were undertaken: (1) Identify the most typical *shapes* (waveforms) that M waves can have and quantify the frequency of appearance of each shape, (2) Identify and discriminate the propagating and non-propagating components of the M wave by examining the changes in the M-wave waveform at different positions along the fibers' direction, (3) Identify and delimitate the propagating and non-propagating components of the M wave by scrutinizing how the different parts of the M wave (positive peak and shoulder) changed with increasing stimulation intensity, and (4) Inquire about the possibility that bipolar M waves also contained non-propagating components.

**Figure 2:**
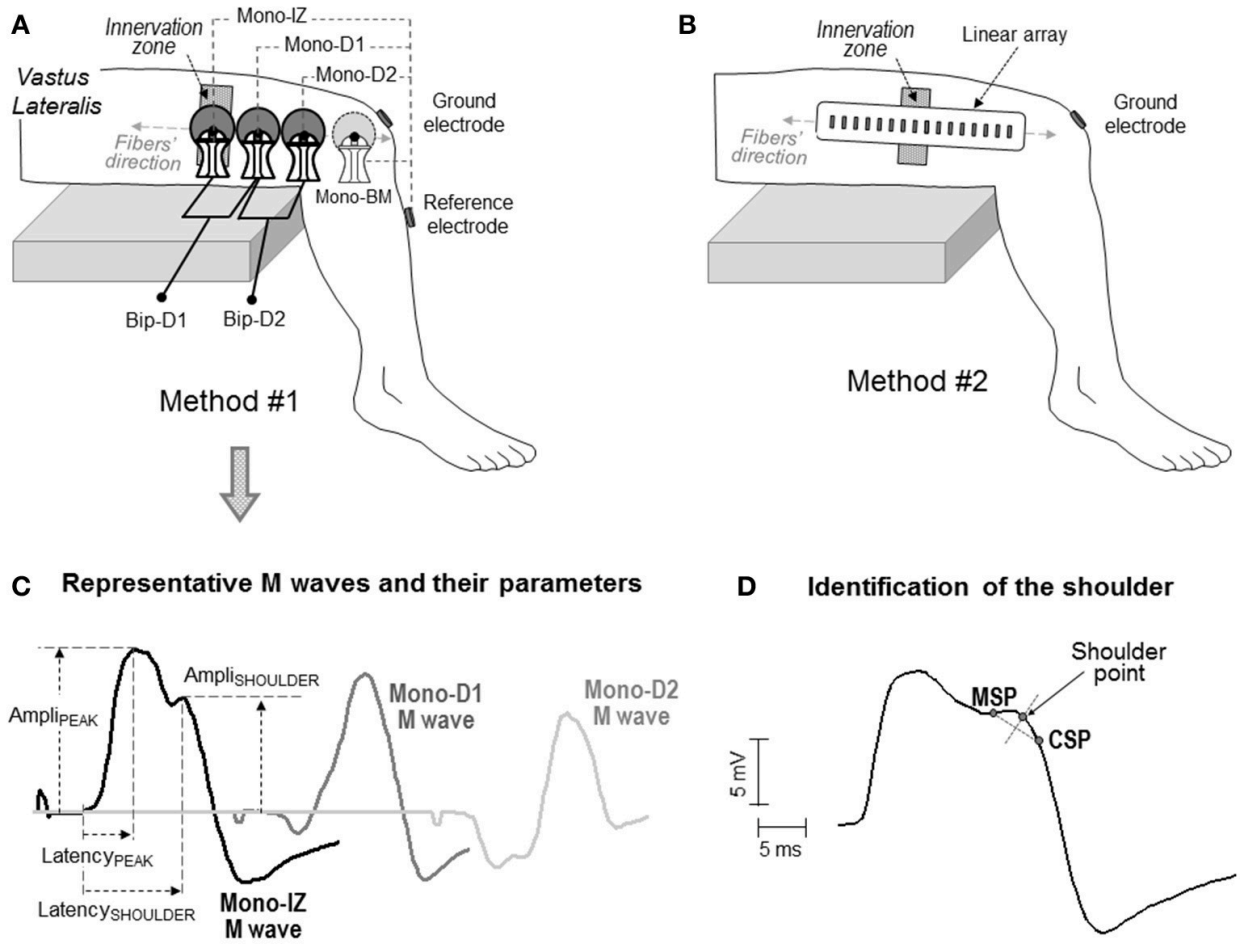
Schematic representation of the two electrode arrangements for EMG recording adopted in the study: one involving large self-adhesive electrodes with a recording diameter of 10 mm **(A)**, and the other consisting of small electrodes (1 × 5 mm) arranged in a linear array **(B)**. In both cases, the electrodes were aligned with the muscle fiber direction. In the first method **(A)**, the proximal electrode was placed above the innervation zone (mono-IZ), whereas the other two electrodes (mono-D1 and mono-D2) were located on the distal side of the innervation zone. In some participants, an additional electrode was placed beyond the muscle (mono-BM). Each of these electrodes was used as the active electrode in the monopolar configuration. The bipolar M waves (Bip-D1 and Bip-D2) were obtained by subtracting monopolar M waves from pairs of adjacent electrodes. **(C)** Definition of parameters in the M waves obtained with the first method: amplitude of the positive peak (Ampli_PEAK_) and shoulder (Ampli_SHOULDER_), and latency of the positive peak (Latency_PEAK_) and shoulder (Latency_SHOULDER_). **(D)** Method for identifying the location of the shoulder point using the minimum slope point (MSP) and the constant slope point (CSP), see text for details.

The above four actions were addressed using two different approaches. The first approach involved the use of the *classical* self-adhesive surface electrodes with a 10-mm recording diameter [(Figure 2A), see below for details]. These electrodes and their arrangement were chosen as they represent one of the most commonly used electrode settings in exercise physiology and clinical disciplines. With these *classical* electrode settings, data were collected from 126 participants who volunteered in previous studies from our group. Participants (110 men, 16 women) were aged between 22 and 40 years (mean ± SD: 27.8 ± 5.6 years). Their average height and weight was 176.3 ± 4.2 cm and 69.5 ± 4.9 kg, respectively. Written informed consent was obtained from the participants of the study.

The second approach involved the use of a linear array of 16 electrodes, each of them having a small recording surface, and separated by a short inter-electrode distance (Figure 2B), see below for details]. The smaller size of the electrodes in the array allowed a more accurate assessment of the changes in the M-wave shape along the fibers' direction, and hence a more reliable identification of the propagating and non-propagating components in the M wave. This precise shape examination was conducted in eight male participants, aged between 22 and 28 years (mean ± SD: 24.9 ± 3.4 years). Their average height and weight was 177.2 ± 4.8 cm and 71.5 ± 5.3 kg, respectively. Written informed consent was obtained from the participants of the study. Approval for the project was obtained from the local Ethics Committee, and all procedures used in this study conformed to the Declaration of Helsinki.

### Anatomical and electrophysiological aspects of the *Vastus Lateralis*

According to anatomical dissection studies, the motor branch of the *vastus lateralis* (which branches out from the femoral nerve trunk) divides into two (sometimes three) primary sub-branches that penetrate the muscle at the proximal (superior sub-branch) and distal (inferior sub-branch) one-third of the *vastus lateralis* (Sung et al., 2003). In agreement, three different motor points (proximal, central, and distal) have been identified in most individuals in the *vastus lateralis* (Botter et al., 2011), which correspond to the proximal, central, and distal partitions of this muscle (Becker et al., 2010). In the present study, the EMG analysis was confined to the distal one-third of the *vastus lateralis* for two reasons. First, as mentioned above, this portion of the muscle is generally innervated by the distal branch, and hence a well-defined innervation zone is likely to be found (Ye et al., 2015). Moreover, the SENIAM guidelines (Hermens et al., 2000) recommend placing the electrodes on this portion of the muscle.

### Identification of the muscle fibers' direction and innervation zone

The procedures to identify the muscle fibers' direction and the innervation zone were similar for the two methods of M-wave detection mentioned above and were performed as follows. Participants were seated comfortably on a custom-built chair with a knee angle of 90° and a trunk-thigh angle of 100°. Extraneous movements of the upper body were limited by two crossover shoulder harnesses and a belt across the lower abdomen. Before testing, the skin over the distal portion of the *vastus lateralis* was prepared by light abrasion with sandpaper and cleaning with rubbing alcohol. Surface EMG signals were recorded with a *dry* linear array of 16 electrodes (5 × 1 mm, 5 mm inter-electrode distance), which was connected to a multichannel amplifier (OT Bioelettronica, Torino; bandwidth 10–500 Hz). EMG signals were detected in single differential configuration. The direction of the muscle fibers was identified by choosing the orientation of the array that leads to the minimal variation in the shape of the action potentials (Farina et al., 2002). A line parallel to the orientation of the muscle fibers was marked on the skin with a waterproof felt-tip pen. The main innervation zone was visually identified by the EMG channel(s) that had minimum amplitude or phase reversal (Masuda et al., 1985).

### Experimental protocol

Two different methods of EMG recording were adopted, involving two different electrode types and settings (Figure 2). In the first method, M waves were recorded using three circular self-adhesive Ag/AgCl electrodes (Kendall Meditrace 100, Tyco, Canada) from 126 participants. The electrodes had a recording diameter of 10 mm and were separated by a distance of 20 mm. Three pairs of electrodes were placed in a belly-tendon configuration, as depicted in Figure 2A. The “belly” electrodes of the pairs were placed along a line parallel to the orientation of the muscle fibers. To standardize the electrode positioning in all participants, the belly electrodes were placed relative to the innervation region as follows. The proximal belly electrode was always located above the innervation zone, and was referred to as mono-IZ. The other two belly electrodes, mono-D1 and mono-D2, were positioned adjacent to each other, on the distal side of the innervation zone, as shown in Figure 2A. For these belly electrodes, the “tendon” (reference) electrodes were placed, close together, over the bony surface of the tibia, and the ground electrode was positioned over the ipsilateral patella. The M waves recorded under this belly-tendon configuration can be considered as monopolar M waves inasmuch as the tendon electrode was placed on an electrically non-active site of the body (Tucker and Türker, 2005). In 13 of the 126 participants assessed with this electrode configuration, an additional monopolar electrode was located beyond the muscle (mono-BM), adjacent to the mono-D2 electrode, and also aligned with the fibers' direction, as shown in Figure 2A. This mono-BM M wave was recorded to assess whether its waveform was a polarity-inverted version of the mono-IZ M wave, as indicated by Lateva et al. (1996).

The electrode settings described above were also used to examine the M waves in bipolar configuration. The bipolar M waves were obtained off-line by subtracting monopolar M waves from pairs of adjacent electrodes. With our electrode settings, the Bip-D1 and Bip-D2 M waves were derived in the proximal-to-distal direction (Figure 2A). These bipolar M waves were examined for the purpose of determining whether or not non-propagating components are not completely canceled out when the proximal and distal monopolar M waves are subtracted.

In the second method of EMG recording, M waves were recorded from eight participants using a 16-channel linear electrode array with small contact surfaces and inter-electrode distances (1 × 5 mm, 5 mm inter-electrode distance, OttinoBioelettronica, Torino, Italy; see Figure 2B). This linear probe was oriented along the muscle fibers' direction, which had been determined previously (see above). The array was located so that its eighth electrode coincided with the position of the innervation zone. To ensure proper electrode–skin contact, electrode cavities of the array were filled with 20–30 μl of conductive paste. The surface EMG signals were amplified, sampled at 2048 Hz, bandpass filtered (3 dB bandwidth, 10–500 Hz), and converted to digital data by a 12-bit A/D converter (EMG-USB, OT Bioelettronica, Torino, Italy). A reference electrode strap (OttinoBioelettronica, Torino, Italy) was moistened and wrapped around the subject's dominant wrist to reduce electromagnetic noise as much as possible.

### Stimulation procedure

M waves were evoked by applying electrical stimuli (single rectangular voltage pulses, 1-ms duration) to the femoral nerve while the quadriceps muscle was at rest using a constant current stimulator (Model DS7AH, Digitimer, Hertfordshare, UK). The cathode was a circular (5-cm diameter) self-adhesive electrode (Dermatrode, American Imex, Irvine, CA, USA), positioned in the femoral triangle, 3–5 cm below the inguinal ligament. The anode consisted of a rectangular (5 × 10 cm) self-adhesive electrode (Compex, Ecublens, Switzerland), placed over the gluteal fold to close the stimulation current loop. Stimulus pulses of increasing intensity were delivered every 20s. Specifically, current intensity was increased in steps of 10 mA, from 0 mA up to the value beyond which there was no further increase in M-wave amplitude. This intensity was termed Imax. Stimulus maximality was further confirmed by increasing stimulation level by 20% and then noticing that M-wave amplitude parameters were the same (Rodriguez-Falces and Place, 2016).

### Data analysis

Data were recorded with a commercially-available software (AcqKnowledge, Biopac Systems, Goleta, CA, USA), and the M waves were monitored online for any abnormality that could be a sign of the electrodes not being properly attached to the skin. Subsequently, data were exported to Matlab (version R2012b; The Math-Works, Natick, MA, USA) for quantitative analysis using ad-hoc scripts. Following conventional standards in Clinical Neurophysiology, M waves were plotted with negative voltages upward. Thus, for simplicity in the present study, the terms “positive” and “negative” were used with reference to the direction on the plot rather than the true electrical polarity.

For the mono-IZ, mono-D1, mono-D2, and mono-BM M waves, the amplitude and latency of the positive peak (Ampli_PEAK_, Latency_PEAK_) were measured (see Figure 2C). In addition, for the mono-IZ M wave, the amplitude and latency of the shoulder (Ampli_SHOULDER_, Latency_SHOULDER_) were computed. The starting points for the Latency_PEAK_ and Latency_SHOULDER_ parameters were determined by a deviation >2 SDs from the baseline harmonic mean. The end points for the Latency_PEAK_ and Latency_SHOULDER_ were set, respectively, at the positions of the positive peak and shoulder of the M wave. Unless otherwise stated, only the most proximal bipolar M wave (Bip-D1) was considered for analysis.

Precise determination of the shoulder-like feature was performed using the slope characteristics of the M wave (i.e., analysis of its first temporal derivative) as shown in Figure 2D. First, the point of smallest slope (MSP) in the descending portion of the first phase was determined. Next, in the portion between the MSP and the M-wave negative peak, we calculated the point at which the slope stabilized at the maximum value (constant slope point, CSP). Third, the straight segment between the MSP and CSP points was created. Finally, the shoulder point was obtained as the intersection with the trace of the M wave of the perpendicular bisector of the MSP-CSP segment.

To obtain an average picture of how M-wave properties changed with stimulation intensity, the stimulation intensities utilized for each participant were first normalized to the range (0–100%, where 100% represents Imax). Then, these normalized recruitment curves were averaged across participants. This processing allowed us to examine the differences in the rate of increase of Ampli_PEAK_ and Ampli_SHOULDER_ with increasing stimulus intensity. For the sake of clarity, only the stimulation intensities of 20, 40, 60, 80, and 100% Imax were considered for statistical analysis.

Data of muscle fiber conduction velocity and fascicle length of the *vastus lateralis* would allow to confirm whether the latency values of shoulder-like feature corresponds to the arrival of the action potentials at the superficial aponeurosis. Estimation of conduction velocity was performed by means of the linear electrode array, using the multidip approach described by Farina and Negro (2007). The multidip method is based on the use of a regression analysis of the spatial and temporal frequencies of multiple dips introduced into the EMG power spectrum through the application of a set of spatial filters (Farina and Negro, 2007). The technique requires four consecutive EMG channels in which a clear propagation of motor unit potentials is seen. In our study, channels 3–6 of the electrode array were chosen. Conduction velocity was estimated during maximal voluntary contractions of 6s, which were performed after the maximal stimulation intensity was found. Three trials, separated by 3 min, were carried out, and the average conduction velocity of the trials were computed.

### Statistics

Kolmogorov-Smirnov tests confirmed that each of the M-wave parameters analyzed in the current study was normally distributed (*P* > 0.05). For the 126 participants assessed with the classical electrodes, differences in the amplitudes and latencies of the M-wave peak and shoulder among the mono-IZ, mono-D1, mono-D2, and mono-BM M waves evoked during maximal stimulation were examined using a one-way ANOVA. To examine differences in the effects of stimulation intensity on the amplitudes of the M-wave peak and shoulder, a two-way repeated-measures ANOVA [relative stimulation intensity (20, 40, 60, 80, and 100% Imax) × amplitude parameter (mono-IZ Ampli_PEAK_, mono-IZ Ampli_SHOULDER_)] was performed. When main effects or interactions were significant, Student-Newman-Keuls *post-hoc* tests for pair wise comparisons were applied. Significance was set at *P* < 0.05. Data were presented as mean ± SD in the text and tables and as mean ± SE in the figures.

## Results

### M-wave analysis using the classical (10-mm recording diameter) electrodes

**Figure 3:**
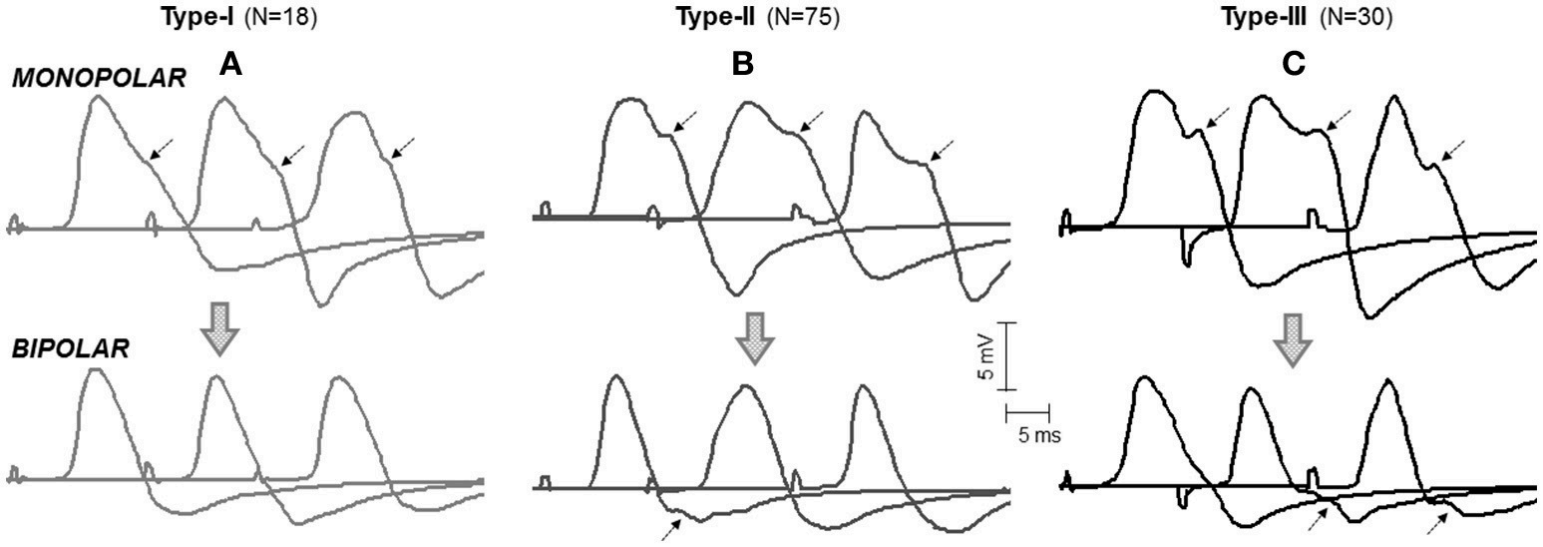
Representative examples of the three types of M waves, type I **(A)**, type II **(B)**, and type III **(C)**, identified in the *vastus lateralis* (over the innervation zone) in monopolar (first row, mono-IZ) and bipolar (second row, Bip-D1) configurations. Each M wave corresponds to a different participant. Note that, for the three morphologies in the monopolar version, the descending portion of the first phase exhibits a brief period of low slope (or shoulder), which is indicated by arrows. For the bipolar M waves, the shoulder lay in the second phase and could not be recognized in all cases; note that the arrows shown on the bipolar M waves have the same latency as the arrows placed in their monopolar counterparts. *N* indicates the number of individuals in each category.

In the vast majority (97%) of the monopolar M waves recorded over the innervation zone (mono-IZ), the descending portion of the first phase did not decrease at a constant rate; rather the decrease was steep at the beginning, and then it slowed down temporarily for a short period, after which the decrease became steep again until the trough (negative peak) of the M wave (for examples, see Figure 3). Hence, the most remarkable common feature of the mono-IZ M waves was a brief period of low slope (or *shoulder*) during the declining first phase. Such *shoulder* could be more or less apparent, and appeared at different heights relative to the first peak. On the basis of the characteristics of the shoulder-like feature, the mono-IZ M waves were roughly classified into three types. Figure 3 shows various representative examples of the type-I, type-II, and type-II waveforms.

In the type-I M waves, the duration of the shoulder was so short that it was visually perceived as a discontinuity point in the slope of the declining first phase (Figure 3A). The frequency of appearance of these M waves was low, representing only 15% of the total. For the type-II M waves, the shoulder was longer, lasting approximately from 1 up to 5 ms (Figure 3B). With this duration, the shoulder was normally recognized as a *plateau* in the middle of the declining first phase. This typology of M wave was the one most frequently found (61%). For the type-III M waves, the shape of the shoulder was different: instead of *plateau* the shoulder adopted the form a hump, or peak (Figure 3C). Thus, type-III M waves displayed two distinct peaks in the first phase. These M waves represented 24% of the total. Of note, some of the bipolar M waves had discontinuities in the slope of their second phase that were similar to the shoulders in the monopolar M waves (Figure 3, second row).

### Identification of propagating and non-propagating components by changing the electrode position

**Figure 4:**
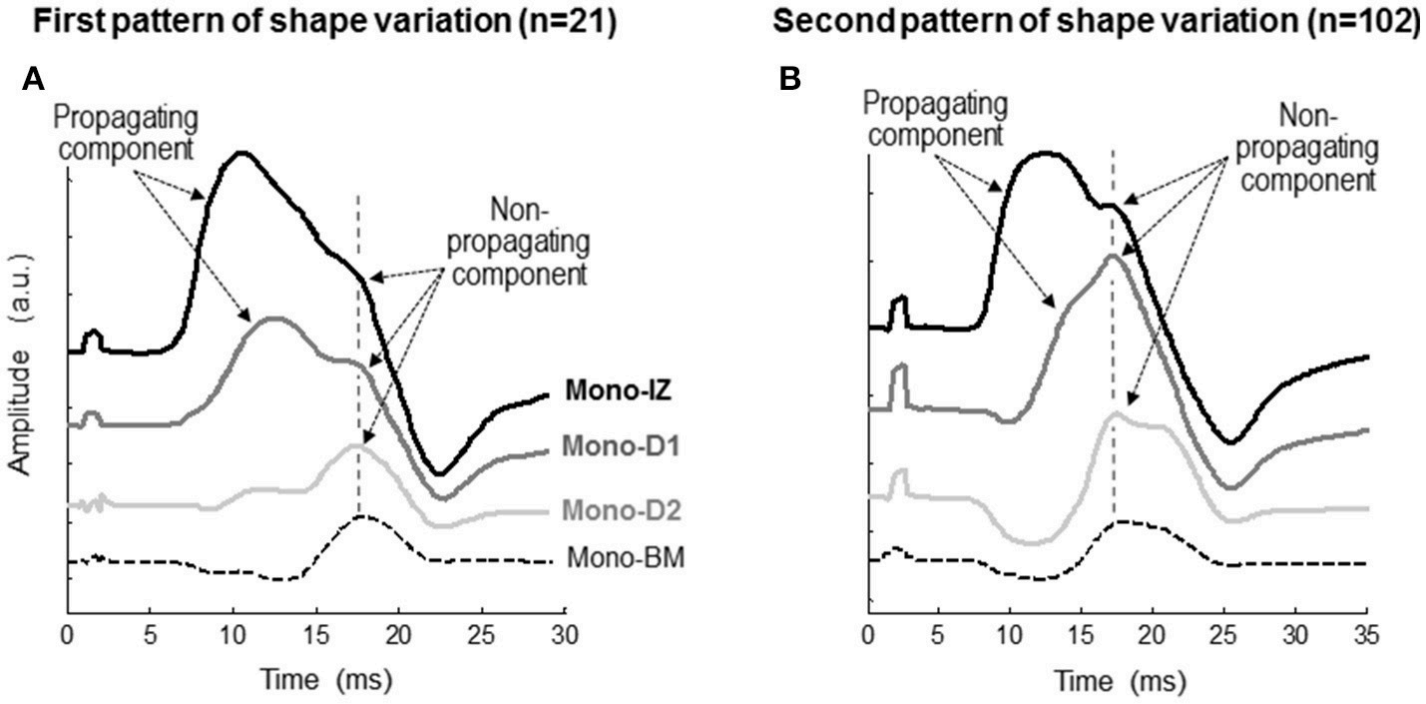
Representative examples of the two patterns of variation, first pattern **(A)**, second pattern **(B)**, in the shape of the monopolar M wave at different recording sites from the innervation zone to the distal tendon of the *vastus lateralis*. Each set of M waves corresponds to a different participant. In each M wave, the propagating and non-propagating components are indicated with arrows. The specific locations of the mono-IZ, mono-D1, mono-D2, and mono-BM electrodes are described in Figure 2. *N* indicates the number of individuals in each category.

To determine the origin of the shoulder-like feature described above, we first identified the propagating and non-propagating components by examining the changes of the M-wave waveform at different recording sites from the innervation zone to the distal tendon (Figure 4). Essentially, two different patterns of changes were observed. In the first pattern (Figure 4A), the overall shape of the M wave did not change significantly between the mono-IZ and mono-D1 locations, and the only difference was that the latency of the first peak increased from the mono-IZ to the mono-D1 M wave, reflecting the propagating character of this peak. Noteworthy, the latency of the shoulder coincided for the mono-IZ and mono-D1 M waves, indicating the non-propagating nature of this feature. In contrast, the shape of the potential changed significantly between the mono-D1 and mono-D2 locations. Indeed, the shoulder of the mono-D1 M wave was transformed into the main positive peak at the mono-D2 position. Finally, the mono-BM M wave had the typical shape of a *beyond-the-muscle* potential: an initial prolonged negative phase, followed by a late positive peak. The above pattern of changes represented only 17% of the total.

In the second pattern of shape variation (Figure 4B), a drastic transformation of the M-wave shape occurred from the mono-IZ to the mono-D1 locations. Specifically, the shoulder of the mono-IZ M wave evolved into the main positive peak of the M wave at the mono-D1 position. In fact, the shoulder of the mono-IZ potential coincided in latency with the positive peak of the mono-D1 and mono-D2 potentials, reflecting the non-propagating nature of these features. As a result of this shape transformation, the rising phase became much more gradual in the mono-D1 than in the mono-IZ M wave. Collectively, the overall shape of the mono-D1 M wave was more similar to that of a *beyond-the-muscle* potential (mono-BM) than to that of an *over-the-innervation-zone* potential (mono-IZ). Indeed, an initial negative phase appeared in the mono-D1 M wave, and the positive peak was principally composed of non-propagating components. The above pattern of changes was the one most commonly found (83%).

**Figure 5:**
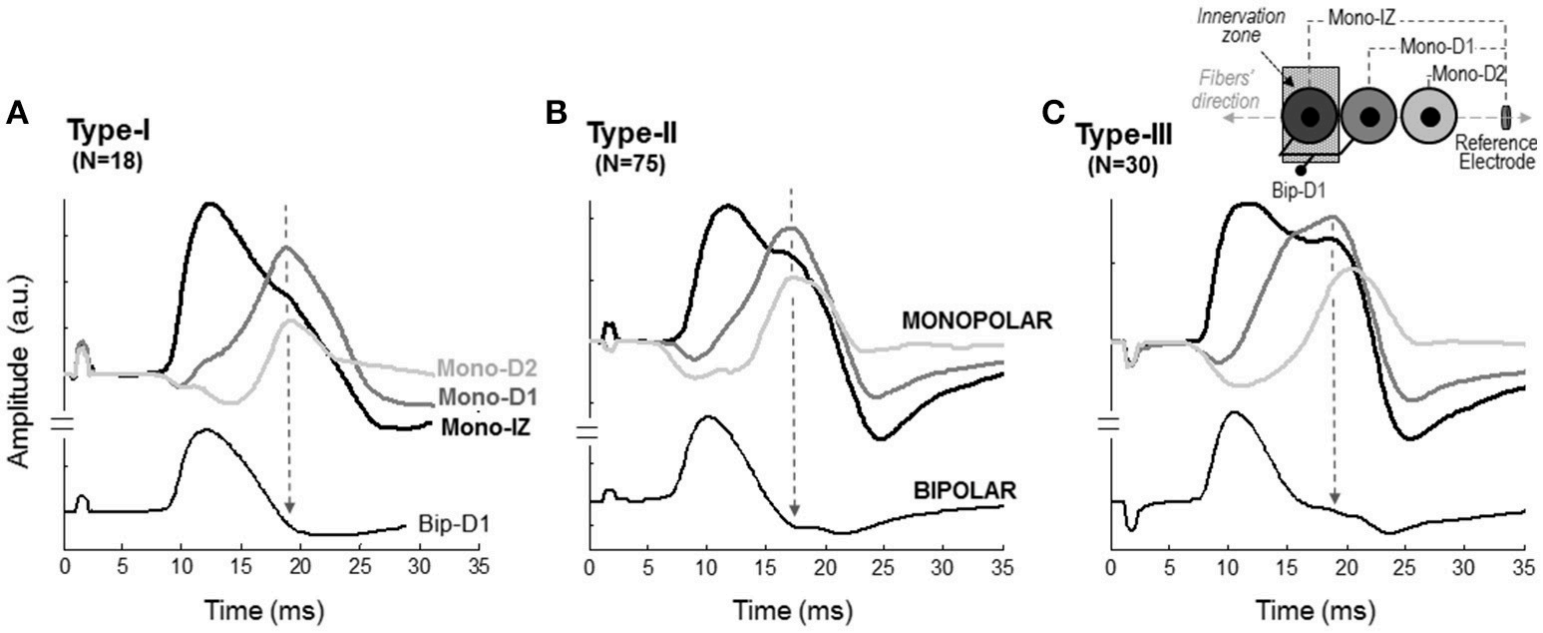
Representative examples of M waves recorded at different positions along the fibers' direction in monopolar (mono-IZ, mono-D1, and mono-D2) and bipolar (Bip-D1) configurations for the type I **(A)**, II **(B)**, and III **(C)** M waves. The specific locations of the mono-IZ, mono-D1, mono-D2, and mono-BM electrodes are described in Figure 2. *N* indicates the number of individuals in each category.

The next step was to verify the correspondence between the latency of the shoulder of the mono-IZ M wave and that of the positive peak of the distal M waves for the whole study group. For clarity, this analysis was performed only for participants demonstrating the second pattern of shape variation (*N* = 102, Figure 5B). Group analysis confirmed that the shoulder of the mono-IZ M wave coincided in latency with the peaks of the mono-D1, mono-D2, and mono-BM M waves (*P* < 0.05, Table 1). Moreover, the alignment of the shoulder of the mono-IZ potential with the positive peaks of the distal potentials was demonstrated separately for the type–I –II, and –III M waves (Table 1), and can be can be visually appreciated in the examples of Figures 5A–C. It must, however, be acknowledged that, in a few cases (8%), the peak of the mono-D2 M wave was slightly shifted to the right in respect to that of the mono-D1 M wave (see Figure 5C).

**Table 1:** Mean (SD) values of the latency and amplitude corresponding to M waves recorded at different positions along the fibers' direction (mono-IZ, mono-D1, mono-D2, and mono-BM) for the different types of M waves (type I, II, and III).

	**Type I (*****N*** = **18)**		**Type II (*****N*** = **75)**		**Type III (** ***N*** = **30)**	
	**Latency (ms)**	**Amplitude (mV)**	**Latency (ms)**	**Amplitude (mV)**	**Latency (ms)**	**Amplitude (mV)**
Mono-IZ M wave (first peak)	6.5(1.4)	12.7(3.1)	6.3(1.3)	12.5(2.2)	6.2(1.8)	12.9(2.1)
Mono-IZ M wave (shoulder)	11.3(3.5)	8.3(2.2)^*^	11.2(2.9)	8.1(2.7)^*^	11.2(3.6)	8.2(2.0)^*^
Mono-D1 M wave (first peak)	11.3(3.3)	9.3(3.4)^*^^†^	11.4(2.8)	8.9(2.8)^*^^†^	11.3(3.3)	9.2(2.5)^*^^†^
Mono-D2 M wave (first peak)	11.5(3.1)	6.5(2.0)^*^^†^	11.6(3.2)	6.3(2.3)^*^^†^	11.6(3.4)	6.2(2.9)^*^^†^
Mono-BM M wave (first peak)	11.8(3.6)	4.3(1.5)^*^^†^	11.7(3.3)	3.9(0.9)^*^^†^	11.6(3.2)	4.2(1.2)^*^^†^

* Indicates significantly different compared to the amplitude of the Mono-IZ peak.

† *Indicates significantly different compared to the amplitude of the shoulder. N indicates the number of individual in each category*.

### Identification of propagating and non-propagating components by changing the stimulation intensity

**Figure 6:**
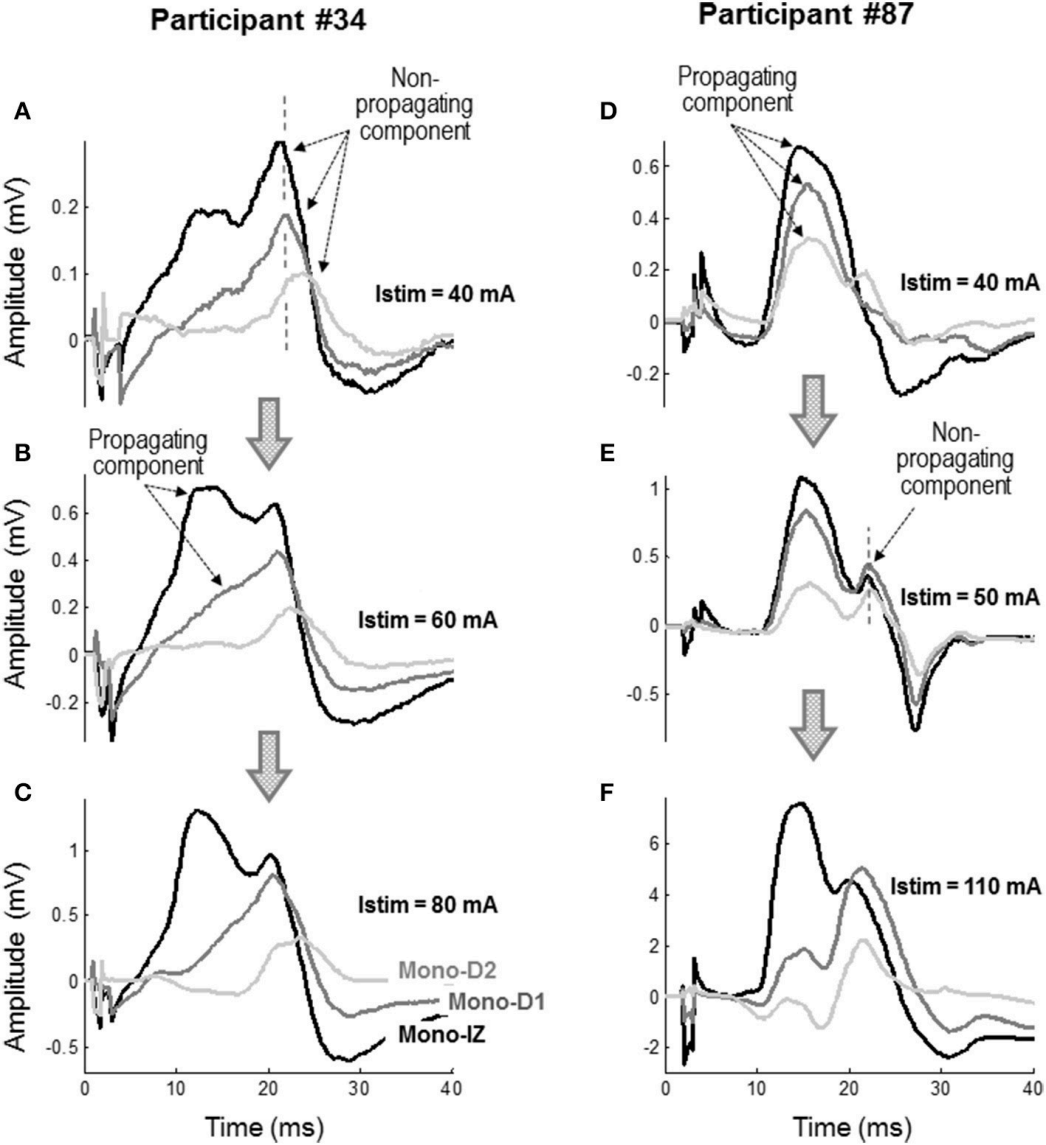
Representative examples of the mono-IZ, mono-D1, and mono-D2 M waves evoked at stimulation intensities (Istim) of 40 mA **(A)**, 60 mA **(B)**, and 80 mA **(C)** for participant #34, and at Istim of 40 mA **(D)**, 50 mA **(E)**, and 110 mA **(F)** for participant #87.

To further confirm the nature of the shoulder-like feature, monopolar M waves were examined at different stimulation intensities to see how propagating and non-propagating components emerged in these M waves with increasing stimulation level. Figure 6 shows two representative examples of the mono-IZ, mono-D1, and mono-D2 M waves evoked at three stimulation intensities for two participants. In the first participant, the three monopolar M waves showed a distinct sharp positive peak with a long latency at Istim = 40 mA (Figure 6A). Because of its long latency and spiky shape, this peak could only be due to action potentials terminating at the aponeurosis. Increasing the stimulation level up to 60 mA resulted in the appearance of a rounded positive peak with shorter latency in the mono-IZ M wave (Figure 6B), which corresponded to the propagating component. In contrast, in the second participant, the three monopolar M waves exhibited a rounded positive peak with short latency, corresponding to the propagating component, at the lowest stimulation intensity (40 mA) (Figure 6D). Increasing the stimulation level up to 50 mA allowed the non-propagating peak to emerge in all three monopolar M waves (Figure 6E). Noteworthy, in the case of the mono-D1 and mono-D2 M waves, the non-propagating peak surpassed the propagating peak when stimulation intensity increased from 50 to 110 mA (Figure 6F).

**Figure 7:**
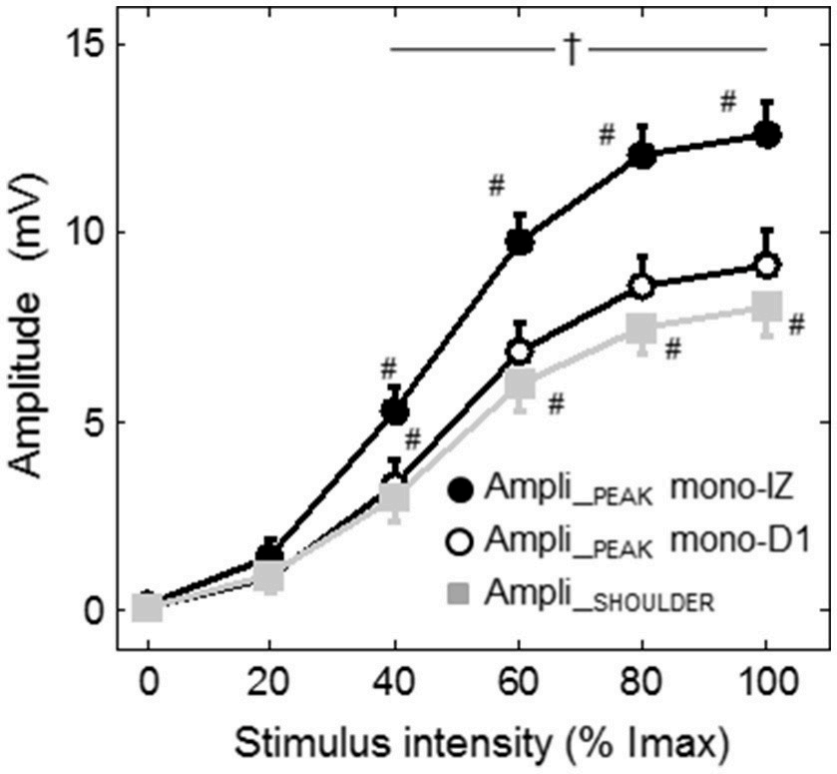
Group data (mean ± SE) of the amplitude of the mono-IZ peak, mono-D1 peak, and shoulder with stimulation intensity plotted as a function of stimulus intensity. ^#^Significantly different from the preceding intensity at *P* < 0.05. ^†^Different between the amplitude of the mono-IZ peak and that of the shoulder and mono-D1 peak at *P* < 0.05. Imax = maximal stimulation intensity.

Figure 7 shows the average increase in the amplitudes of the mono-IZ peak, mono-D1 peak, and shoulder with stimulation intensity for the whole study group. In this figure it can be seen that the increase in amplitude with stimulus intensity was significantly steeper for the mono-IZ peak than for the shoulder and the mono-D1 peak (significant signal type × stimulation intensity interaction, *P* < 0.001). Moreover, the amplitude of the mono-IZ peak was greater than that of the shoulder and the mono-D1 peak over the stimulation range 40–100% Imax (*P* < 0.05).

### Identification of the non-propagating components in bipolar M waves

We also inquired about the possibility that bipolar M waves contained non-propagating components. This possibility was explored using various methods. First, visual inspection of the bipolar M waves revealed that, in 46% of the cases, a discontinuity appeared in the slope of the second phase of the potential, close to the negative peak (Figure 3, second row). Moreover, the discontinuity in the bipolar M wave corresponded in latency with the shoulder of the mono-IZ M wave: this correspondence is evidenced by the vertical dashed lines of Figure 5. Then, in the case of bipolar M waves, the non-propagating components were confined to the second phase of the potential. To further confirm the presence of end-of-fiber components in bipolar M waves, we also examined whether the non-propagating components of M waves recorded by the mono-IZ, mono-D1, and mono-D2 electrodes had different amplitudes. The pooled data of Table 1 indicated that the amplitude of the mono-D1 peak was significantly greater than the shoulder of the mono-IZ M wave and than that of the mono-D2 peak (*P* < 0.05).

### M-wave analysis using the linear electrode array

**Figure 8:**
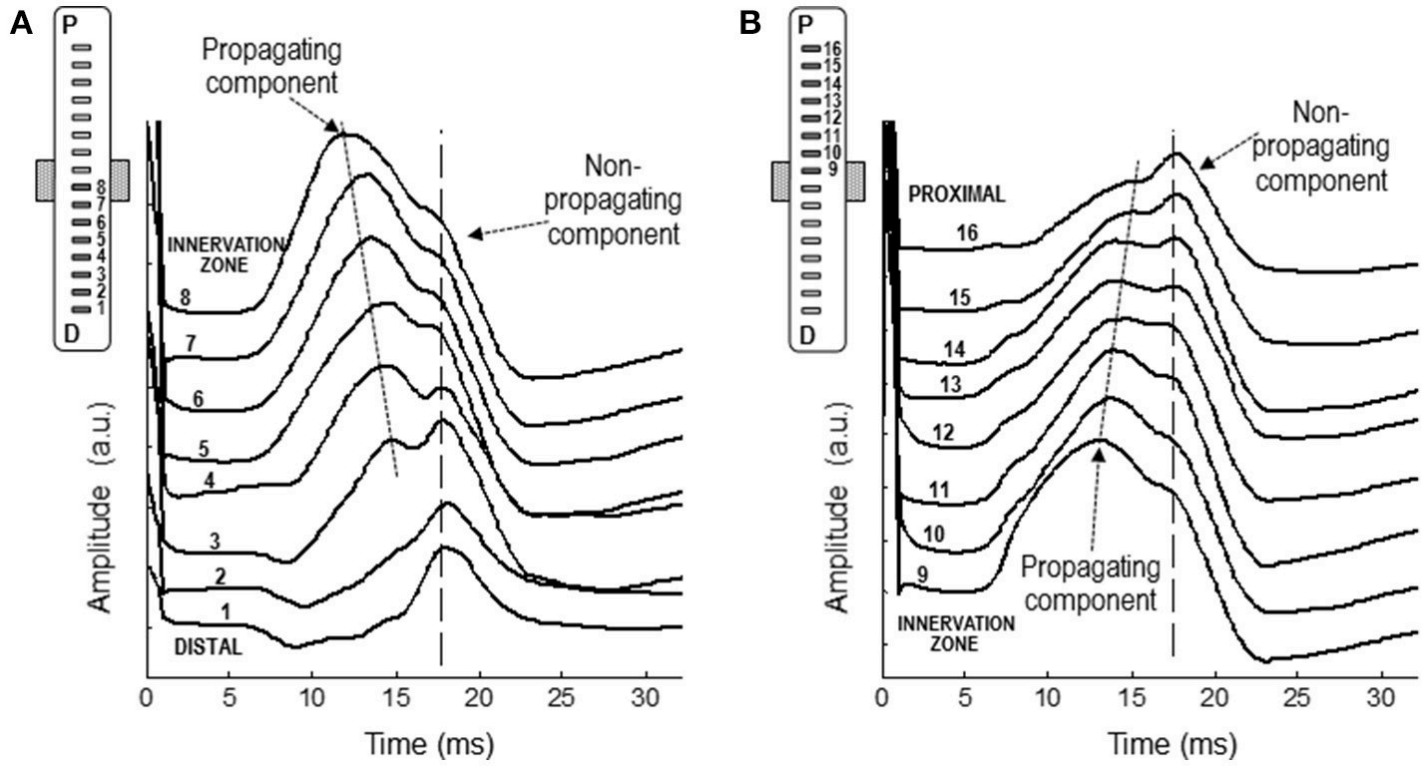
Representative examples of M waves recorded with the linear electrode array at different positions along the muscle fibers' direction, from the innervation zone to the distal **(A)** and proximal **(B)** tendons. In each set of M waves, the propagating and non-propagating components are indicated with arrows.

Figure 8 shows a representative example of the monopolar M waves recorded with the linear electrode array at different positions along the muscle fibers' direction. In the set of M waves recorded from the innervation zone to the distal tendon (Figure 8A), it can be readily seen that latency of the first main peak increased as the M wave is detected further from the innervation zone, from channel 8 to 4, evidencing the propagating nature of this peak. In contrast, the latency of the shoulder was approximately the same across these locations (channels from 8 to 4). It can be also appreciated that the shoulder of the M wave, present in the channels close to the innervation zone, was gradually transformed into the main positive peak as the M wave was detected more distally (channels 3, 2, and 1). Moreover, the latency of the shoulder in the channels close to the innervation zone was similar to that of the positive peak in the distal channels, confirming the non-propagating nature of this feature. The same observations can be made from the set of M waves recorded from the innervation zone to the proximal tendon (Figure 8B). The latency values of the positive peak and shoulder were similar to those obtained using the classical electrodes.

### Estimation of muscle fiber conduction velocity

The linear electrode array was also used to estimate the muscle conduction velocity during maximal voluntary contractions. The mean value of conduction velocity was 4.9 ± 0.7 m/s.

## Discussion

The present study aimed to elucidate the electrical origin of the shoulder-like feature that can be recognized in the descending portion of the first phase of M waves recorded above or close to the innervation zone. The main findings were the following: First, the shoulder was clearly identified in the vast majority (97%) of monopolar M waves, whereas it was less frequently observed (46%) in bipolar M waves. Second, our experiments involving M-wave recordings at different positions along the fibers' direction showed that the shoulder is a non-propagating feature which had the same latency at different distances from the innervation zone. Third, the amplitude of the main positive peak and the amplitude of the shoulder increased at different rates when stimulation intensity was gradually increased. Consideration will first be given to the electrical origin and characteristics of the shoulder-like feature. Subsequently, we will discuss the importance and practical implications of the present findings for the assessment of muscle membrane excitability.

### The shoulder-like feature in monopolar and bipolar M waves

We found that, for the vast majority of the monopolar M waves recorded over the innervation zone (97%), the descending portion of the first phase showed a brief period of low slope, a feature referred to as *shoulder*. This morphologic feature, however, was much less frequently observed in the M waves recorded in bipolar mode (only in 46% of the cases). In addition, the shoulder always appeared in the first phase for monopolar M waves, whilst, for bipolar potentials, it was usually located in the second phase. Moreover, whereas the shoulder was easily recognized in most monopolar M waves, it was not so apparent and well-defined in bipolar M waves. Besides, in some bipolar M waves, the shoulder appeared so close to the negative peak that it could be taken for a second peak (Figures 3B, 5B). This blurred and inconsistent manifestation of the shoulder in the bipolar M waves is due to the signal cancelation occurring in bipolar configuration (see Figure 1C), which removes part of the electrical activity generated by the propagation and extinction of the action potential (Tucker and Türker, 2005). This explains why, in those muscles where the bipolar arrangement is normally adopted, such as the knee extensors, the presence of the shoulder has been overlooked and/or disregarded. On the contrary, the shoulder is commonly observed in other muscles in which the M waves are generally recorded in monopolar configuration, such as the *tibialis anterior* (Thomas et al., 1989; Rodriguez-Falces et al., 2015).

### The electrical origin of the shoulder-like feature

Our results provide several pieces of evidence that the shoulder-like feature of the M wave is due to the termination of the action potential at the superficial aponeurosis. First, M-wave recordings made with the linear array oriented along the fibers' direction showed that the shoulder had the same latency at different longitudinal distances from the innervation zone (Figure 8), thus proving its non-propagating nature. This finding is supported by the recordings made with the classical (10-mm recording diameter) electrodes placed along the fibers' direction, which showed that the shoulder of the mono-IZ M wave coincided in latency with the positive peak of the mono-D1 and mono-D2 M waves (Figures 4, 5). Finally, and perhaps more convincingly, the shoulder of the mono-IZ M wave had the same latency as the positive peak of the *beyond-the-muscle* (mono-BM) M wave. Since the mono-BM potential was recorded beyond the end of the muscle, its terminal positive peak cannot be due to activity propagating under the electrode and can only be the result of the extinction of action potentials.

There are additional (indirect) lines of evidence supporting the non-propagating character of the shoulder. In our experiments involving a gradual increase of the stimulation intensity we found that, for the mono-IZ M wave, the amplitudes of the main positive peak and that of the shoulder increased at different rates (Figure 7), which reflect the different electrical content and nature of these two parts of the M wave. Moreover, the values of latency found for the shoulder (around 11–12 ms) are consistent with the time taken for the action potential to reach the ends of the fibers, as derived from our measurements of muscle fiber conduction velocity and the length of the muscle fibers in the *vastus lateralis* (see below for details).

### Consistency between the estimations of muscle conduction velocity and the latency of the shoulder

To further confirm that the shoulder-like feature of the M wave corresponds to the arrival of the action potentials at the superficial aponeurosis, it is necessary to have an estimate of muscle conduction velocity and fascicle length. Our estimated values of muscle conduction velocity with the linear array (4.9 ± 0.7 m/s) were similar to those measured by previous authors in the *vastus lateralis* using the same technique (Spairani et al., 2012; Butugan et al., 2014; Cadore et al., 2014). On the other hand, estimates of fascicle length of the *vastus lateralis* at a knee joint angle of 90° ranged from 90.2 mm (Kubo et al., 2014) to 110.3 mm (Ando et al., 2016), with most authors providing estimates in the middle of this range (100.6 mm, Fukutani and Kurihara, 2015). Thus, assuming that neuromuscular junctions are located in the middle of the inclined muscle fibers, and hence a fiber semilength of 45–55 mm, this implies that action potentials would take 9.2–12.7 ms to reach the aponeurosis. This propagation time is in close accordance with the values of shoulder latency found here (11.1–11.8 ms).

### The shape and electrogenesis of the M wave are highly sensitive to positional changes of the electrode

We found that the shape of monopolar M waves was highly sensitive to changes of the electrode longitudinal position in respect to the innervation region. Indeed, in nearly 80% of the participants, a displacement of 20 mm from the center of innervation zone in the distal direction was sufficient to induce a deep transformation of the M-wave waveform shape (see Figures 4, 5, 8). More importantly, not only the shape, but also the electrical origin and character of the positive phase of the M wave changed markedly as a result of an electrode shift of 20 mm. Specifically, with the electrode placed over the innervation zone, the positive phase of the M wave was mainly formed of propagating components (excluding the shoulder), whereas 20 mm away from the innervation region, the positive phase was essentially composed of non-propagating components. Therefore, there was a narrow spatial range around the innervation zone (± 20 mm) within which the positive phase of monopolar M waves truly reflected electrical activity propagating under the electrode.

### The importance of the shoulder-like feature for the study of muscle membrane excitability

The ability to identify and discriminate between the parts of the M wave corresponding to the propagation and extinction events is of major importance, as only the propagating portion of the compound potential can be expected to reflect reliably the changes in membrane excitability (Rodriguez-Falces and Place, 2017). It must be stressed that identification of the portion of the M wave with pure, genuine propagating character is difficult, as there is always some degree of overlapping between the propagating and non-propagating components. Yet, the shoulder-like feature represents a simple and unequivocal landmark to distinguish the portion of the M wave that can be considered as being essentially composed of propagating components.

### Practical implications and recommendations

Several important implications can be derived from the present findings. The implications concern M waves recorded in both monopolar and bipolar configurations. First, it has been demonstrated that, in monopolar mode, end-of-fiber signals contaminate not only the second phase of the M wave, but also the final portion of the first phase. This implies that only the portion of the first phase comprised between the onset and the shoulder-like feature can be regarded as having a propagating character and, therefore, only this initial part of the M wave is valid for the study of muscle membrane excitability. Second, we found that, only when the electrode is located within ± 20 mm around the innervation zone, does the positive phase of a monopolar M wave have a propagating character. This constraint is relevant for studies where the M wave is used as an indicator of sarcolemmal excitability, as it implies that, if the belly-tendon configuration is used, the belly electrode must necessarily lie above or close to the innervation zone. This recommendation of placing the active electrode above the innervation region had already been followed in the past by several authors in the *biceps brahii* (Cupido et al., 1996), *tibialis anterior* (Thomas et al., 1989), and *abductor pollicis* (Bigland-Ritchie et al., 1982), thereby indirectly supporting our results. Furthermore, the choice of the innervation zone as the preferred site for electrode location in monopolar configuration has been further legitimized by a recent study (Rodriguez-Falces, 2016).

It is commonly assumed that end-of-fiber signals can be reduced by means of appropriate spatial filters (Disselhorst-Klug et al., 1997). Moreover, it has been proposed that the single-differential (bipolar) configuration is sufficient to remove almost completely non-propagating components (van Vugt and van Dijk, 2001). However, the present study has shown that, at least for M waves recorded bipolarly in the *vastus lateralis* using the classical pair of electrodes with a 20-mm inter-electrode distance, the second (negative) phase most likely contain non-propagating components. The reason is that the end-of-fiber potentials detected at two positions separated by 20 mm in the muscle fibers' direction differ in amplitude and shape (Rodriguez-Falces and Place, 2014). As a result, when the proximal and distal monopolar M waves are subtracted to form the bipolar M wave, there will not be perfect cancelation of their respective non-propagating components. The results of the present study all point to this conclusion. First, in 46% of the bipolarly-recorded M waves, a distinct shoulder appeared in the second (negative) phase, which is indicative of the presence of non-propagating components. Moreover, the latency of the shoulder of the bipolar M wave coincided with the latency of the mono-IZ M wave. Therefore, we propose that in the *vastus lateralis*, even in bipolar configuration, only the first phase of the M wave genuinely reflects propagation of action potentials along the fibers; the second phase is likely contaminated by the presence of end-of-fiber signals.

## Conclusions

In conclusion, for the majority (97%) of the M waves recorded monopolarly over the innervation zone of the *vastus lateralis*, the descending portion of the first phase showed a brief period of low slope, a feature referred to as shoulder. This feature was much less frequently observed (46%) and less pronounced in M waves recorded bipolarly due to signal cancelation. Examination of M waves at different positions along the fibers' direction revealed that the shoulder is a non-propagating feature, which has the same latency at different distances from the innervation zone. These experiments provided convincing evidence that the shoulder-like feature is the electrical manifestation of the termination of the action potential at the superficial aponeurosis of the *vastus lateralis*. The following implications are drawn. For an M wave to have a positive phase mainly formed by propagating components, it is necessary that the electrode is located within ± 20 mm around the innervation zone. In monopolar M waves, only the initial portion of the first phase, comprised between the M-wave onset and the shoulder, genuinely reflects propagation of the action potential along the fibers. In bipolar M waves, however, the whole first phase has a pure propagating character, while the second phase is likely contaminated by the extinction of action potentials. We conclude that, only the amplitude of the first phase, and not the second, of M waves recorded monopolarly and/or bipolarly above (or in close proximity to) the innervation zone can be used reliably to monitor possible changes in muscle membrane excitability.

## Author contributions

JR-F and NP: Substantial contributions to the conception or design of the work; acquisition, analysis, and interpretation of data for the work; drafting the work or revising it critically for important intellectual content; final approval of the version to be published; agreement to be accountable for all aspects of the work in ensuring that questions related to the accuracy or integrity of any part of the work are appropriately investigated and resolved.

### Conflict of interest statement

The authors declare that the research was conducted in the absence of any commercial or financial relationships that could be construed as a potential conflict of interest.
